# Cross-metathesis of polynorbornene with polyoctenamer: a kinetic study

**DOI:** 10.3762/bjoc.11.195

**Published:** 2015-10-01

**Authors:** Yulia I Denisova, Maria L Gringolts, Alexander S Peregudov, Liya B Krentsel, Ekaterina A Litmanovich, Arkadiy D Litmanovich, Eugene Sh Finkelshtein, Yaroslav V Kudryavtsev

**Affiliations:** 1 Topchiev Institute of Petrochemical Synthesis, Russian Academy of Sciences, Leninsky prosp. 29, 119991 Moscow, Russia; 2 Nesmeyanov Institute of Organoelement Compounds, Russian Academy of Sciences, Vavilova str. 28, 119991 Moscow, Russia; 3 Chemistry Department, Moscow State University, Leninskie gory 1, build. 3, 119991 Moscow, Russia

**Keywords:** cross-metathesis, 1^st^ generation Grubbs’ catalyst, interchange reactions, kinetics, multiblock copolymer

## Abstract

The cross-metathesis of polynorbornene and polyoctenamer in *d*-chloroform mediated by the 1^st^ generation Grubbs’ catalyst Cl_2_(PCy_3_)_2_Ru=CHPh is studied by monitoring the kinetics of carbene transformation and evolution of the dyad composition of polymer chains with in situ ^1^H and ex situ ^13^C NMR spectroscopy. The results are interpreted in terms of a simple kinetic two-stage model. At the first stage of the reaction all Ru-benzylidene carbenes are transformed into Ru-polyoctenamers within an hour, while the polymer molar mass is considerably decreased. The second stage actually including interpolymeric reactions proceeds much slower and takes one day or more to achieve a random copolymer of norbornene and cyclooctene. Its rate is limited by the interaction of polyoctenamer-bound carbenes with polynorbornene units, which is hampered, presumably due to steric reasons. Polynorbornene-bound carbenes are detected in very low concentrations throughout the whole process thus indicating their higher reactivity, as compared with the polyoctenamer-bound ones. Macroscopic homogeneity of the reacting media is proved by dynamic light scattering from solutions containing the polymer mixture and its components. In general, the studied process can be considered as a new way to unsaturated multiblock statistical copolymers. Their structure can be controlled by the amount of catalyst, mixture composition, and reaction time. It is remarkable that this goal can be achieved with a catalyst that is not suitable for ring-opening metathesis copolymerization of norbornene and *cis*-cyclooctene because of their substantially different monomer reactivities.

## Introduction

A desired sequence of monomer units in a polymer chain can be achieved not only in the course of polymerization but also through chemical modification of macromolecules [1]. In particular, main-chain polyesters and polyamides are capable of cross-reactions (also known as interchange reactions) characterized by the rearrangement of macromolecular backbones via break up and the formation of new C–O and C–N bonds [2]. Such reactions are extensively used in practice for combining the functionality and the processability of different polymers in one material [3]. A more recent line of research is associated with dynamic covalent polymers containing alkoxyamine, imine, disulfide, and other easily cleavable moieties in their backbone [4–5]. It aims at stimuli-responsive, intelligent polymeric materials, the structure and properties of which can be precisely controlled by adjusting temperature, pH or by introducing low molecular additives.

Much less is known about the possibility of monomer unit reshuffling in unsaturated carbon-chain polymers, such as polydienes, which constitute a core of commercially available elastomers. As soon as the olefin metathesis was discovered, it became possible to think on the implementation of cross-reactions between C=C bonds in polymers. Until recently the studies were focused on the intramolecular reactions [6–7] and polymer degradation by interaction with olefins [8–9], whereas the interchain cross-metathesis was merely an idea for many years [10]. Only recently a few publications appeared that demonstrated the possibility of using the Grubbs’ Ru catalysts to make polybutadiene networks malleable [11] and self-healing [12] and to marry chain-growth 1,4-polybutadiene with step-growth unsaturated polyesters [13–14]. Hydrogenation of the reaction product led to saturated ethylene/ester copolymers with a multiblock chain structure predefined at the cross-metathesis stage [14].

In our previous communication [15] we reported the obtaining of a copolymer of norbornene (NB) and *cis*-cyclooctene (COE) by the cross-metathesis of polynorbornene (poly(1,3-cyclopentylenevinylene), PNB) with polyoctenamer (poly(1-octenylene), PCOE). It is noteworthy that the reaction is readily mediated by the 1^st^ generation Grubbs’ catalyst Cl_2_(PCy_3_)_2_Ru=CHPh (Gr-1), which is not suitable for metathesis ring-opening copolymerization of NB and COE. Our approach makes it possible to synthesize statistical multiblock NB-COE copolymers containing up to 50% of alternating dyads. By adjusting the conditions of the cross-metathesis between PNB and PCOE, such as the polymer/catalyst ratio, PNB/PCOE ratio and their molecular masses, reaction time, etc., one can obtain NB-COE copolymers with the mean block lengths varying from 200 to 2 units.

It is noteworthy that PNB and PCOE are commonly synthesized by ring-opening metathesis polymerization (ROMP). PNB is a well-known commercial product available under the trademark Norsorex^®^ [8,16], which is mainly used as a solidifier of oil and solvent for the complete absorption of oil or other hydrocarbons. PCOE, known as Vestenamer^®^ [17], is a semicrystalline rubber applied as a polymer processing aid for extrusion, injection molding etc. Though easily homopolymerized, NB and COE hardly enter metathesis copolymerization [18–19] because of the much higher activity of NB possessing a considerably more strained bicyclic structure, which gets opened during ROMP [8,20]. To solve this problem, two approaches were elaborated in the literature. One approach utilizes a specially designed catalyst that facilitates the formation of a highly alternating NB-COE copolymer [21–25]. The other approach is associated with a reduction of the polymerization activity of NB through introducing substituents into its molecule [26–28]. Therefore, the cross-metathesis of PCOE and PNB can be considered as a novel way to statistical NB-COE copolymers.

In the present article we try to gain more insight into this reaction by undertaking a kinetic study. We begin with discussing the choice of the reaction media and solution properties of PCOE, PNB, and their mixture in CHCl_3_ studied by light scattering. Then we describe use of the in situ ^1^H NMR spectroscopy for monitoring the separate reactions between Gr-1 and PCOE and between Gr-1 and PNB in CDCl_3_. This technique is widely applied for investigating ROMP in the presence of well-defined catalysts since it allows quantitative determination of the active complex type and conversion during the reaction [29–31]. By fitting the experimental data with a simple kinetic model we estimate and compare the formation and decay rates of Ru–carbene complexes bound to PCOE and PNB. Then we proceed to the investigation of PCOE/PNB/Gr-1 mixtures, where we combine in situ ^1^H NMR measurements of the concentrations of Ru–carbene complexes with ex situ ^13^C NMR measurements of alternating dyad content in the NB-COE copolymer. Such dyads are formed via the reactions of PNB-bound carbenes with COE units and, vice versa, of PCOE-bound carbenes with NB units. The above kinetic model for the separate reactions of PCOE and PNB with Gr-1 is extended, which makes it possible to outline the scenario of the cross-metathesis of those polymers in the presence of the Gr-1 catalyst.

## Results and Discussion

The initial homopolymers, PCOE and PNB, were synthesized by the ROMP of COE and NB, respectively, using Gr-1 under the conditions that prevent the formation of cyclooligomers (at a high monomer concentration). As known from the literature [29], Gr-1 cannot initiate a living process of COE and NB so that the obtained polymers are rather polydisperse because of back-biting and chain-transfer reactions (the molar-mass dispersity *Ð* is close to 2 for PCOE and to 3 for PNB). For more details on the polymer synthesis and characterization, see the Experimental section.

### Light-scattering studies on PCOE and PNB solutions

First of all, it was important to find a suitable solvent that provides homogeneity of the reaction media. Chloroform (CHCl_3_ or CDCl_3_) was chosen as the best solvent for PCOE/PNB mixtures compared with toluene, THF, CH_2_Cl_2_, and PhCl. Since we are interested in the cross-metathesis, the polymer concentration in solution should be as high as possible to minimize the impact of intrachain reactions [7]. At the same time increasing polymer concentration can lead to polymer/solvent and (in mixtures) polymer/polymer phase separation. We addressed this issue with the light scattering measurements on PCOE (*M*_n_ = 140000 g/mol, *Ð* = 1.9), PNB (*M*_n_ = 80000 g/mol, *Ð* = 2.8), and PCOE/PNB solutions in CHCl_3_.

**Figure 1:**
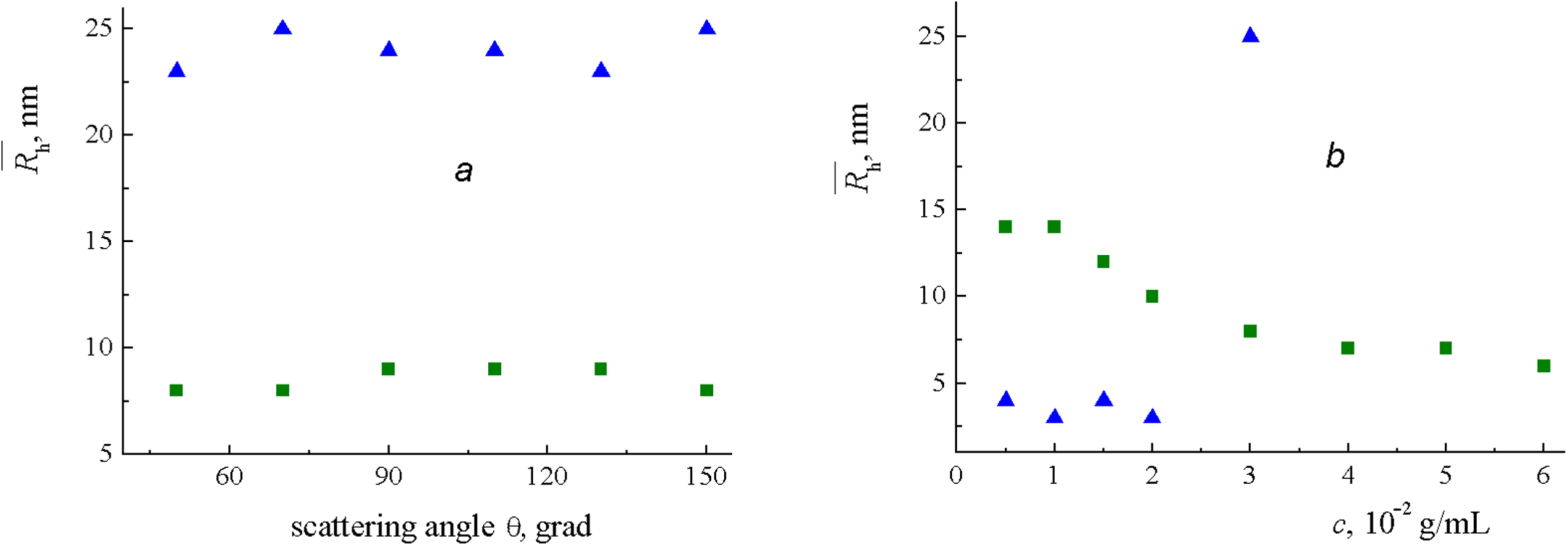
Dependences of the (blue) PCOE and (green) PNB mean hydrodynamic radius 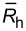 in CHCl_3_ on the (a) light scattering angle θ at *c* = 0.03 g/mL and (b) concentration *c* at θ = 150° found by DLS at 25 °C.

For both polymers, only one relaxation mode was observed. The mean hydrodynamic radius 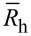 calculated from its relaxation rate was independent of the light scattering angle (Figure 1a). This proves the diffusive nature of the concentration relaxation processes in the studied solutions. Therefore, the concentration dependence of 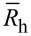 was measured at a maximum available angle of θ = 150°, where the contribution of dust particles to scattering is minimized. As seen from Figure 1b, PNB demonstrated the typical concentration behavior for a polymer in good solvent [32]. In the dilute regime (*c* < 0.01 g/mL) 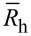 = 14 nm characterizes the mean size of a polymer coil. At higher concentrations macromolecules overlap and their self-diffusion is replaced with a faster cooperative diffusion. In that case 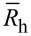 slowly decreases with *c* corresponding to a distance at which hydrodynamic interactions are screened out. For the PCOE solution Figure 1b displays a quite different concentration dependence of 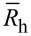. In the dilute regime flexible PCOE macromolecules form very compact coils of 4 nm size, which are much smaller than those of rigid PNB chains of nearly the same *M*_w_. At *c* = 0.03 g/mL 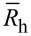 is abruptly increased, thus indicating the aggregation of PCOE chains into particles of 25 nm mean size. At even higher concentrations, DLS measurements with PCOE are impossible since the solution is not filterable through a 220 nm porosity membrane. Taking into account that the melting temperature of PCOE is about 45 °C, we can relate aggregation in the PCOE solutions at 25 °C to the onset of crystallization. In any case, it makes no sense to carry out metathesis reactions at a PCOE concentration higher than 0.03 g/mL.

**Figure 2:**
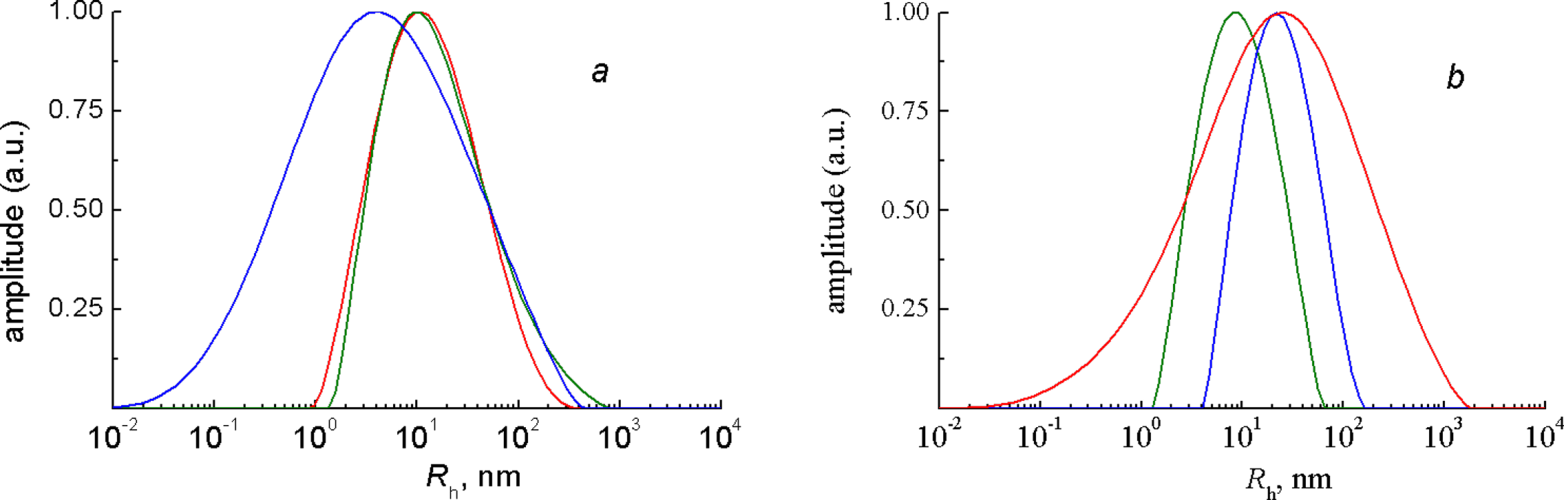
Hydrodynamic radius distributions (normalized by their maximum values) in the CHCl_3_ solutions of (blue) PCOE, (green) PNB, and (red) their mixture at the concentration of (a) 0.015 g/mL and (b) 0.03 g/mL of each of the polymers measured by DLS at θ = 150° and 25 °C.

DLS experiments on the PCOE/PNB mixtures were conducted at the equal component concentrations taken to be 0.015 and 0.03 g/mL. Figure 2 compares the normalized hydrodynamic radius distributions in the separate components and in their mixture. It is seen that the (mixture) red and (PNB) green curves in Figure 2a almost coincide, which means that the concentration relaxation at lower concentrations is controlled by larger PNB particles (at the concentration of 0.015 g/mL they may be still identified with the individual macromolecules). In the more concentrated solution (Figure 2b) PCOE particles grow (see also Figure 1b), thereby increasing the mean hydrodynamic radius of the PCOE/PNB mixture to 25 nm. It is important that in the both cases the mixture displays a unimodal distribution indicating that no polymer/polymer segregation takes place.

The data of static scattering shown in Table 1 corroborate this conclusion because the mean intensity of light scattered by the mixture with the total polymer concentration of 0.06 g/mL appear, on the one hand, approximately equal to the sum of intensities produced by the solutions of the pure components of that mixture and, on the other hand, nearly twice as much as the intensity of light scattered by the mixture with the total concentration of 0.03 g/mL. Thus, PCOE/PNB solutions in CHCl_3_ with the concentration of each component close to 0.03 g/mL can be considered as suitable objects for studying cross-metathesis reactions.

**Table 1:** Static scattering intensity from different CHCl_3_ solutions:

Solute	Polymer concentration, g/mL	Scattering intensity, counts s^−1^

PCOE	0.03	1940
PNB	0.03	3170
PCOE/PNB	0.03	2590
PCOE/PNB	0.06	5070

### Interaction of the Gr-1 catalyst with PCOE and PNB

**Figure 3:**
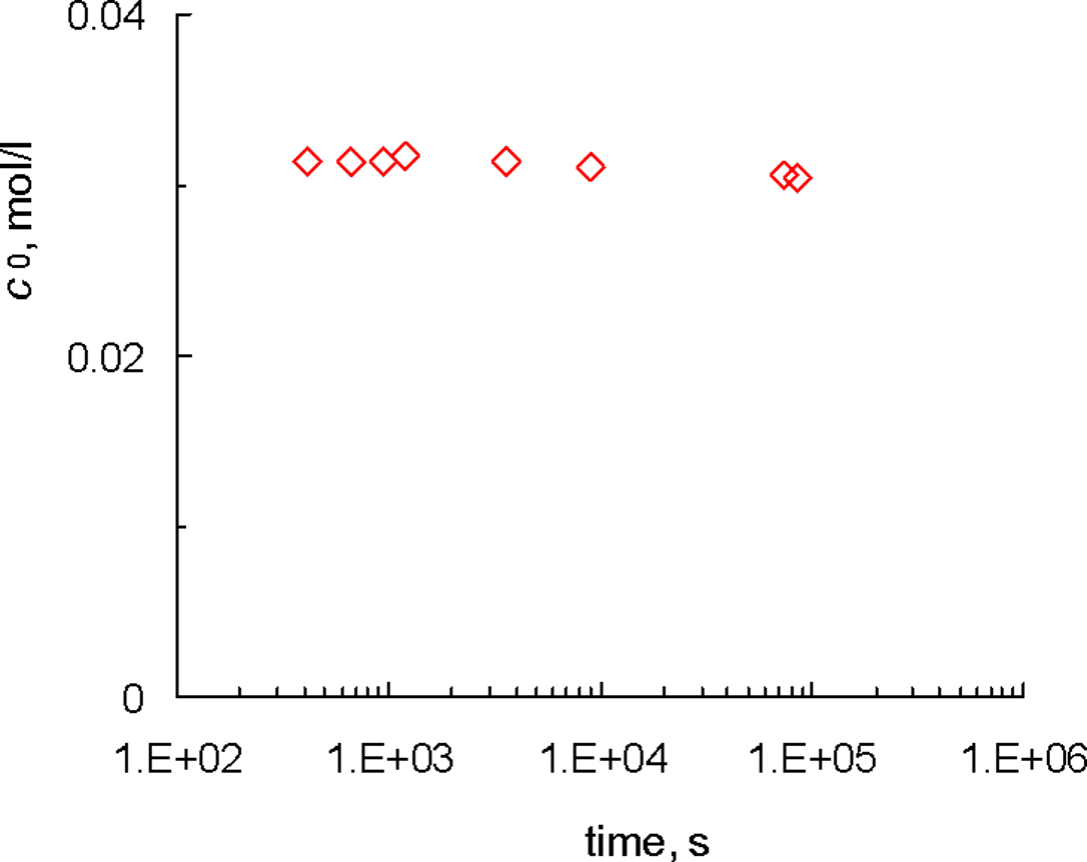
Stability of the primary carbene [Ru]=CHPh in the pure solvent (CDCl_3_).

Dissolving Gr-1 in CDCl_3_ results in the formation of a product, which we call a primary [Ru]=CHPh carbene. Its ^1^H NMR spectrum is characterized by a peak at 20.0 ppm. Figure 3 demonstrates that in the absence of polymers a 0.03 M solution of Gr-1 in CDCl_3_ is practically stable at 20–25 °C during one day, which is a characteristic timescale in our further experiments. The decrease in the primary carbene concentration *c*_0_ does not exceed 3%, being within the accuracy of the NMR method. Thus we can neglect the decay of primary carbenes due to the reasons other than their interaction with macromolecules.

**Scheme 1:**
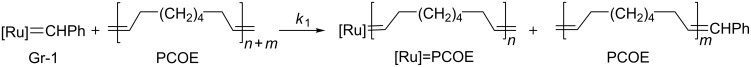
Formation of polyoctenamer-bound carbene by the interaction of Gr-1 with PCOE.

Interaction of PCOE (*M*_n_ = 120000 g/mol, *Ð* = 1.8) with Gr-1 was studied in CDCl_3_ at the initial polymer/catalyst concentration ratio of 20:1. Note that the initial catalyst concentration found by in situ NMR was somewhat lower in all our experiments and these effective values were used in the kinetic calculations. Along with the singlet at 20.0 ppm the ^1^H NMR spectrum showed a new peak at 19.3 ppm, which grew rapidly to 40% of the initial primary carbene within 5 min of the reaction. According to the accepted mechanism of olefin metathesis mediated by Gr-1 [30], this signal can be attributed to a new, secondary carbene ([Ru]=PCOE) formed via break up of a PCOE chain attacked by a primary carbene, as shown in Scheme 1. The mixture viscosity was considerably reduced at the early stage of the reaction (10–20 min) indicating a decrease in the molar mass of PCOE due to its interaction with Gr-1. Looking ahead, we note that similar effects were observed for PNB and PCOE/PNB solutions interacting with this catalyst.

**Figure 4:**
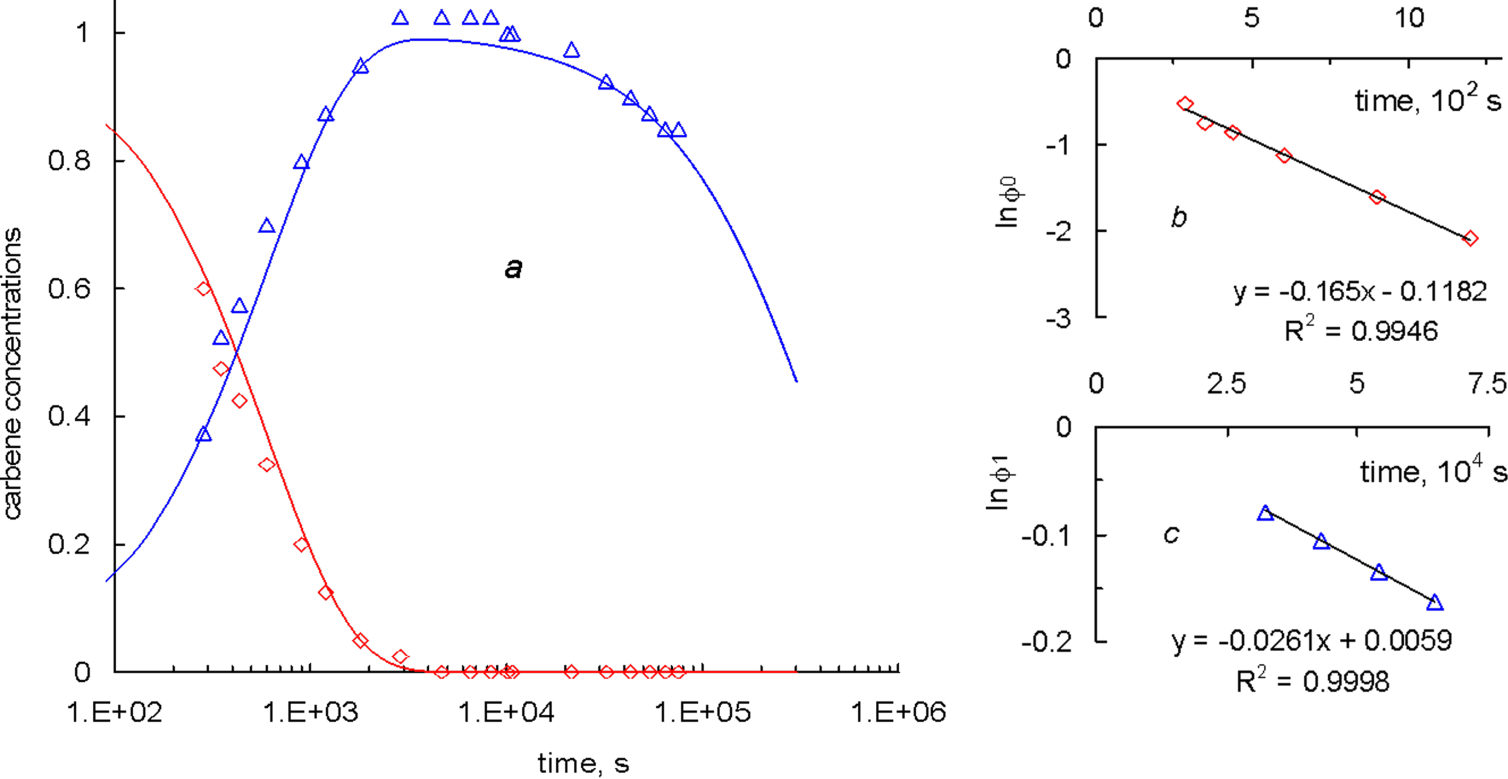
(a) Dependences of the normalized (red) [Ru]=CHPh and (blue) [Ru]=PCOE carbene concentrations on time: (points) experimental data, (curves) calculations according to Equation 1 with the rate constants *k*_1_ = 3.1 × 10^−3^ L mol^−1^ s^−1^ and *k*_1d_ = 2.6 × 10^−6^ s^−1^ found from the (b) early and (c) late kinetic stages of the reaction.

After 1 h the primary carbene signal almost disappeared, while that of the [Ru]=PCOE carbene reached its maximum, kept constant for a couple of hours, and then began to decline very slowly, while the molar mass of the system remained approximately constant after an initial drop. The dependences of the (*c*_0_) [Ru]=CHPh and (*c*_1_) [Ru]=PCOE carbene concentrations, normalized by the initial value *c*_0_(*t* = 0) = *c*_in_, on time are shown as points in Figure 4a. The observed fast transformation of the primary carbenes into the secondary ones followed by the slow decay of the latter can be described in terms of a simple kinetic model.

Let us introduce the rate constants *k*_1_ and *k*_1d_ characterizing two mentioned processes. The first of them is a reversible reaction but this can be neglected due to a considerable excess of the polymer with respect to the catalyst (the repeating unit concentration *c*_p_ = 0.532 mol/L >> *c*_in_ = 0.0213 mol/L). According to the literature data [30], the carbene decay can proceed either as a first-order or second-order reaction. The latter option implies coupling of two polymer chains through the reaction between their end groups, which would lead to an increase in the average molar mass of the polymer. Monitoring the molar mass distribution by GPC does not reveal such effect, therefore, the decay of [Ru]=PCOE carbenes can be described as a first-order reaction with the rate proportional to the carbene concentration. Thus, the concentrations of the primary and secondary carbenes are described by the following equations

[2]
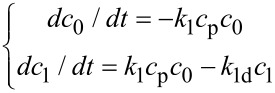


with the initial conditions *c*_0_(*t* = 0) = *c*_in_, *c*_1_(*t* = 0) = 0.

At a constant polymer concentration *c*_p_ = const, the solution of Equation 2 reads

[1]
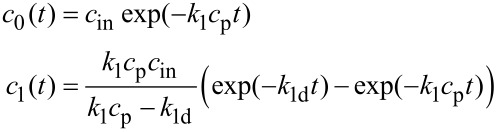


Since [Ru]=CHPh carbenes are completely converted into [Ru]=PCOE ones long before the carbene decay becomes noticeable, then *k*_1_*c*_p_ >> *k*_1d_ and, therefore, these constants can be found separately by representing the early and late kinetic data in the semi-logarithmic coordinates of Figure 4b and Figure 4c. These plots are obviously linear that yields *k*_1_*c*_p_ = 1.65 × 10^−3^ s^−1^ (so that *k*_1_ = 3.1 × 10^−3^ L mol^−1^ s^−1^) and *k*_1d_ = 2.6 × 10^−6^ s^−1^. Red and blue lines in Figure 4a correspond to the *c*_0_(*t*)/*c*_in_ and *c*_1_(*t*)/*c*_in_ dependences calculated from Equation 1 with the above found values of *k*_1_ and *k*_1d_. Close fitting of the experimental data corroborates the consistency of our kinetic approach.

**Scheme 2:**
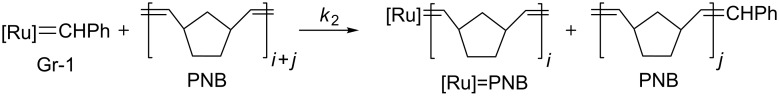
Formation of polynorbornene-bound carbene by the interaction of Gr-1 with PNB.

Interaction of PNB (*M*_n_ = 60000 g/mol, *Ð* = 2.6) with Gr-1 was studied in a similar way. In that case new resonances in the ^1^H NMR spectrum (18.82, 18.83, 18.94 ppm) appeared only after several minutes of the reaction. It can be identified as a secondary [Ru]=PNB carbene formed via cleavage of a PNB chain under the action of a primary carbene, as shown in Scheme 2.

**Figure 5:**
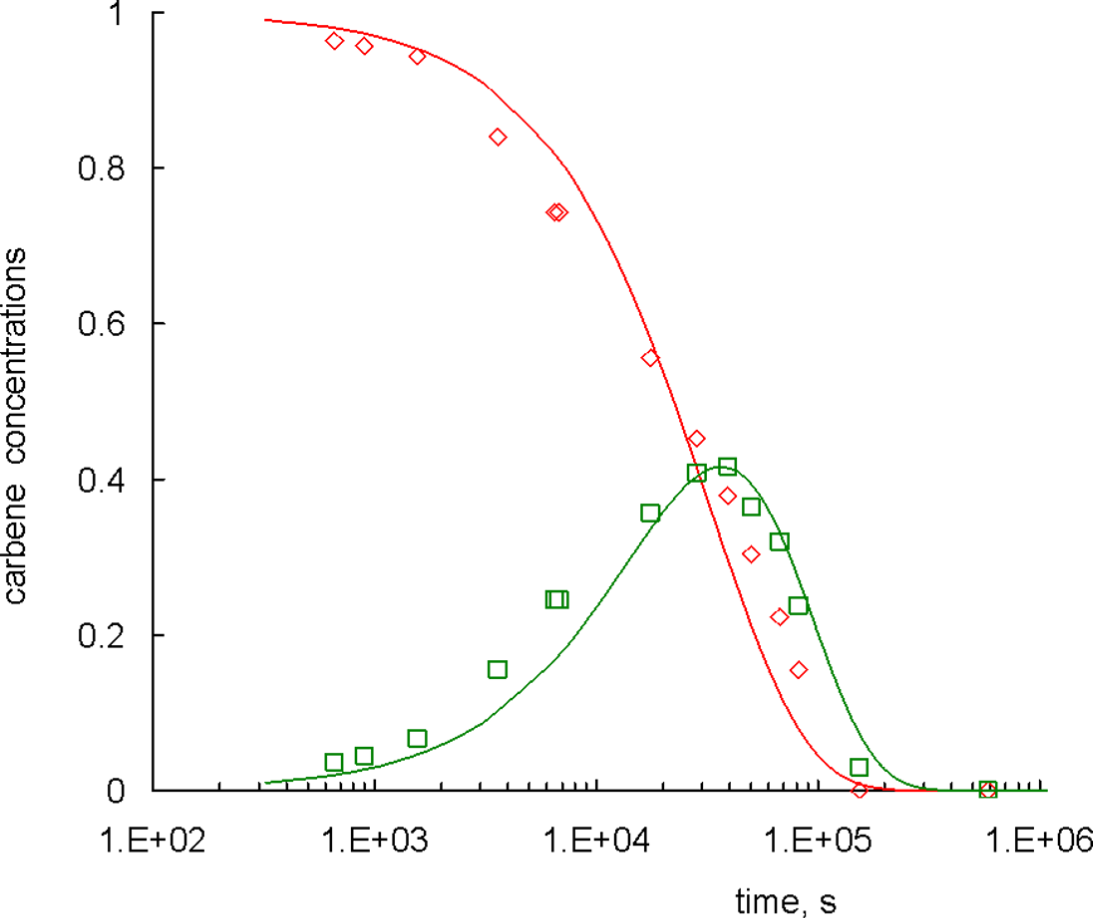
(a) Dependences of the normalized (red) [Ru]=CHPh and (green) [Ru]=PNB carbene concentrations on time: (points) experimental data, (curves) calculations according to Equation 3 with the rate constants *k*_2_ = 5.4 × 10^−5^ L mol^−1^ s^−1^ and *k*_2d_ = 2.4 × 10^−5^ s^−1^.

After 1 h only about 20% of the primary carbenes were transformed into secondary ones. The concentration of [Ru]=PNB carbenes reached its maximum at ca. 11 h from the outset of the reaction and immediately began to decline. The dependences of the (*c*_0_) [Ru]=CHPh and (*c*_2_) [Ru]=PNB carbene concentrations, normalized by the initial value *c*_0_(*t* = 0) = *c*_in_, on time are shown as points in Figure 5. The peak value of *c*_2_ constitutes only 40% of *c*_in_, which means that the processes of the secondary carbene formation and decay cannot be separated in the time scale of our experiment.

Nevertheless, we tried to describe the experimental data with the model introduced above. A solution of the kinetic equations for this case is given by the expressions

[3]
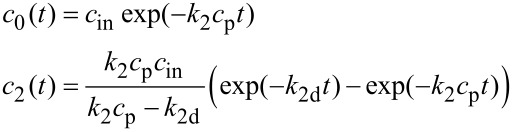


that are similar to Equation 1 up to replacing *k*_1_ with *k*_2_, *k*_1d_ with *k*_2d_, and *c*_1_ with *c*_2_, *c*_p_ = 0.575 mol/L.

The rate constant *k*_2_ was found by fitting the whole *c*_0_(*t*) curve to the experimental data, whereas for *k*_2d_ we focused on the position and value of the maximum of the *c*_2_(*t*) curve. As seen from Figure 5, the agreement between the model and experiment is not as good as for PCOE even for the best fit (*k*_2_ = 5.4 × 10^–5^ L mol^–1^ s^–1^, *k*_2d_ = 2.4 × 10^–5^ s^–1^). The reason of this discrepancy is not clear taking into account a very standard dynamical behavior of PNB solutions in the DLS experiments reported above. We supposed that it could be correlated with a high viscosity of the PNB solution at early stages of the reaction, which was decreased rather slowly due to lower activity of the primary carbene, as compared with the PCOE case. However, when we synthesized PNB (*M*_n_ = 28000 g/mol, *Ð* = 2.8) of nearly half the molar mass of the first sample, the two-constant kinetic model gave approximately the same performance.

In any case we can firmly conclude that *k*_1_ >> *k*_2_. In other words, the Gr-1 catalyst bounds to PCOE chains much more easily than to PNB ones. We can speculate that this property is correlated with the volume of groups surrounding double C=C bonds, i.e., it is sterically caused by more bulky groups in PNB chains that effectively hinder the attack of Gr-1. At the same time, we find that *k*_1d_ << *k*_2d_, which means that [Ru]=PNB carbenes are considerably less stable than [Ru]=PCOE ones, which are in turn inferior to the primary [Ru]=CHPh carbenes in the absence of polymers. With that notion we turn to studying chemical transformations in a PCOE/PNB mixture in the presence of Gr-1 catalyst.

### Cross-metathesis in the mixture of PCOE and PNB

**Scheme 3:**
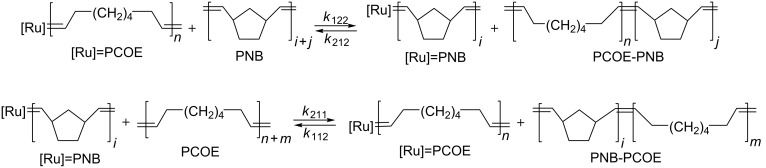
Elementary cross-metathesis reactions in the mixture of PCOE with PNB.

Interaction of PCOE (*M*_n_ = 142000 g/mol, *Ð* = 1.9), PNB (*M*_n_ = 60000 g/mol, *Ð* = 2.6), and Gr-1 was studied in CDCl_3_ solution at the initial concentration ratio [PCOE]/[PNB]:[Gr-1] = 10:10:1 (mol/mol). The chosen total polymer concentration of 4–6% (wt/v) was a compromise between being well above the crossover concentration in order to study the law of mass action kinetics and restricting aggregation of PCOE chains detected by DLS. We supposed that, apart from the reactions of polymer carbenes formation shown in Scheme 1 and Scheme 2 above and their decay, the cross-metathesis reactions could take place as depicted in Scheme 3.

**Figure 6:**
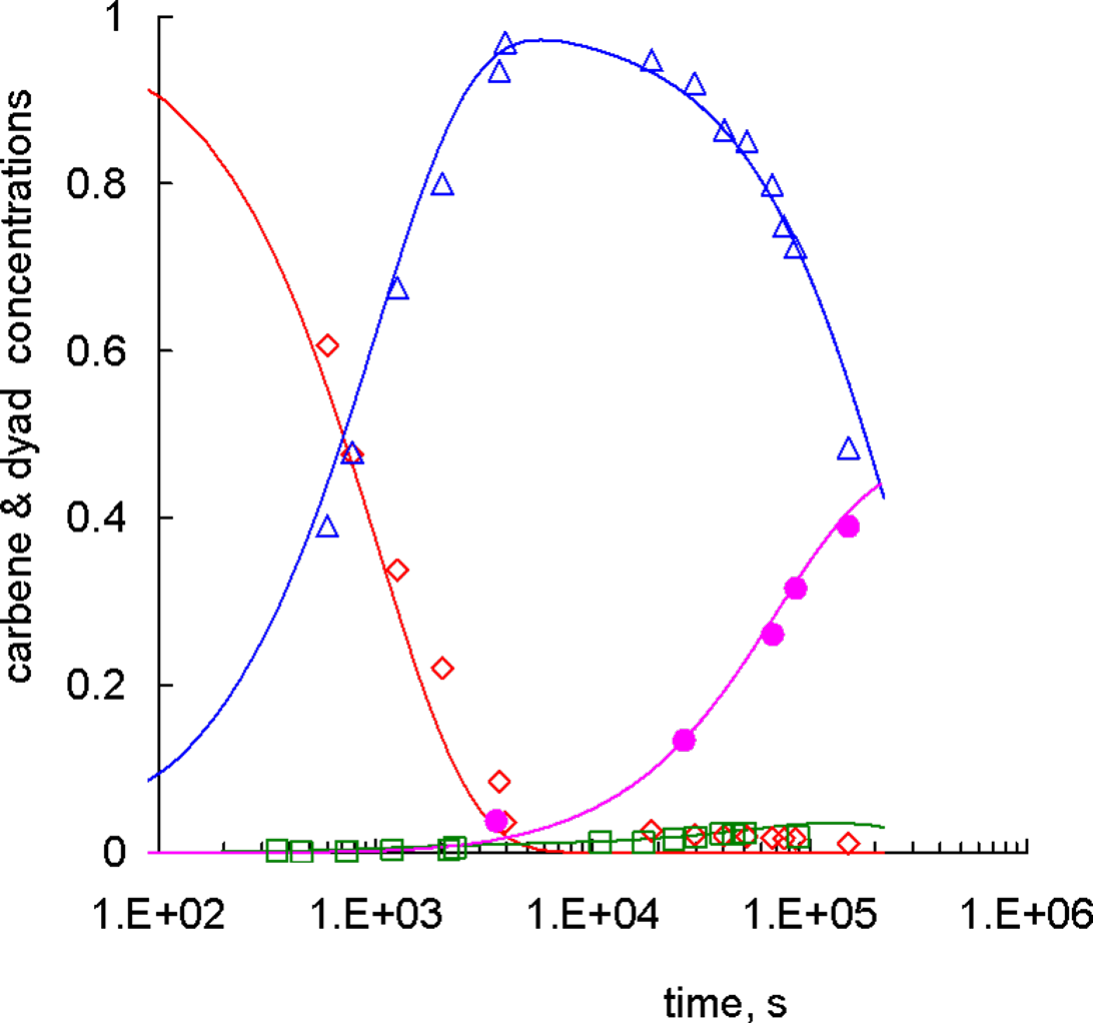
Dependences of the normalized (red) primary, (blue) PCOE, and (green) PNB carbene concentrations and (purple) the fraction of alternating NB-COE dyads on time: (points) experimental data, (curves) calculations according to Equation 4 with the rate constants *k*_1_ = 3.1 × 10^−3^ L mol^−1^ s^−1^, *k*_2_ = 0, *k*_1d_ = 2.6 × 10^−6^ s^−1^, *k*_2d_ = 2.4 × 10^−5^ s^−1^, *k*_211_ = 2.2 × 10^−2^ L mol^−1^ s^−1^, *k*_122_ = *k*_211_/100, *k*_212_ = *k*_112_ = (*k*_211_*k*_122_)^1/2^.

However, two orders of magnitude difference in the activity of Gr-1 with respect to PCOE and PNB left little chance to observe the formation of [Ru]=PNB carbenes in the equimolar mixture. In situ experiments on a 600 MHz NMR spectrometer allowed detecting this secondary carbene at 18.82–18.94 ppm, but its concentration throughout the reaction was indeed very low, as shown by the green squares in Figure 6. One could guess that if [Ru]=PCOE were the only active polymer carbene, then the extent of the cross-metathesis would be very low and the fraction of alternating NB-COE dyads in a copolymer product would be limited by the initial catalyst/polymer concentration ratio of 1/20. Nevertheless, ex situ ^13^C NMR experiments demonstrated that the alternating dyads shown with the full purple circles in Figure 6 not only appeared but gradually became to prevail in the NB-COE copolymer.

This fact can be understood if we assume that the concentration of [Ru]=PNB carbenes is low because they actively react with PCOE (the second direct reaction of Scheme 3), being an important intermediate in the cross-metathesis between PCOE and PNB. Indeed, the reactants here are a [Ru]=PNB carbene that decays faster than a [Ru]=PCOE one and a PCOE chain that is attacked by Gr-1 easier than a PNB one. Therefore, it will be not surprising if this reaction is characterized by the highest reaction rate of four elementary processes depicted in Scheme 3.

The kinetic equations describing reactions in the mixture under study are written down in Equation 4.

[4]
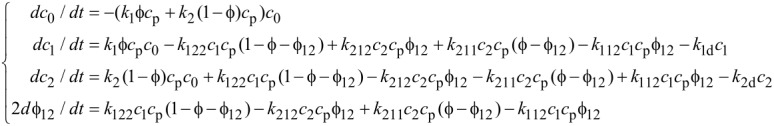


In Equation 4 *c*_0_, *c*_1_, *c*_2_ are the concentrations (mol/L) of [Ru]=CHPh, [Ru]=PCOE, and [Ru]=PNB carbenes, respectively; 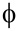 and 2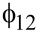 are the molar fractions of PCOE units (which is constant) and alternating (COE-NB and NB-COE) dyads. Note that Equation 4 implies that the law of mass action is valid and does not discriminate between interchain and intrachain reactions. The initial conditions for it read

[5]
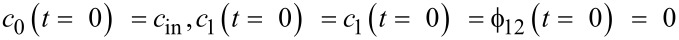


where the initial carbene concentration *c*_in_ = 0.0266 mol/L is again assumed to be much less than the total polymer concentration *c*_p_ = 0.586 mol/L.

Values of the rate constants *k*_1_, *k*_2_, *k*_1d_, and *k*_2d_ can be taken from the above considerations of PCOE – Gr-1 (*k*_1_ = 3.1 × 10^−3^ L mol^−1^ s^−1^ and *k*_1d_ = 2.6 × 10^−6^ s^−1^) and PNB – Gr-1 (*k*_2_ = 5.4 × 10^−5^ L mol^−1^ s^−1^ and *k*_2d_ = 2.4 × 10^−5^ s^−1^) reactions. Looking ahead, we should note that nothing is changed if we just put *k*_2_ to zero, which means that [Ru]=PNB carbenes are formed via the cross-metathesis reaction rather than by the direct transformation of primary [Ru]=CHPh carbenes.

There are still four rate constants (*k*_122_, *k*_212_, *k*_211_, and *k*_112_, where the first index denotes the type of an interacting polymer-bound carbene and the last two indices designate the type of a dyad containing a reacting C=C bond) unknown and only one “new” 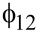
(*t*) function available for fitting. Therefore we will search for the highest rate constant *k*_211_ that describes the attack of a [Ru]=PNB carbene onto a PCOE chain, as discussed above. We also assume that the rate constant *k*_122_ responsible for the interaction of a [Ru]=PCOE carbene with a PNB chain is a hundred times smaller than *k*_211_, by analogy with ca. hundred times smaller reaction rate of Gr-1 with PNB than that of Gr-1 with PCOE. In this manner we take into account the difference in the local environment of C=C bonds in PNB and PCOE. The remaining two constants describing the interaction of [Ru]=PCOE and [Ru]=PNB carbenes with NB-COE heterodyads are taken to be equal to each other and to the geometric mean of *k*_122_ and *k*_211_: *k*_212_ = *k*_112_ = (*k*_211_*k*_122_)^1/2^, since a C=C bond in a NB-COE dyad should be more accessible than in a NB-NB dyad but less than in a COE-COE dyad.

With these assumptions made, we achieved a good agreement between the dependences *c*_0_(*t*)/*c*_in_, *c*_1_(*t*)/*c*_in_, *c*_1_(*t*)/*c*_in_, and 2
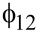
(*t*) calculated for *k*_211_ = 2.2 × 10^−2^ L mol^−1^ s^−1^ and the corresponding experimental NMR data plotted in Figure 6. Qualitatively, it means that the cleavage of a polymeric double C=C bond is about an order of magnitude more probable in the reaction with a polymer-bound Ru-carbene than with a [Ru]=CHPh carbene. Further kinetic studies on that issue are needed to get quantitative results.

**Figure 7:**
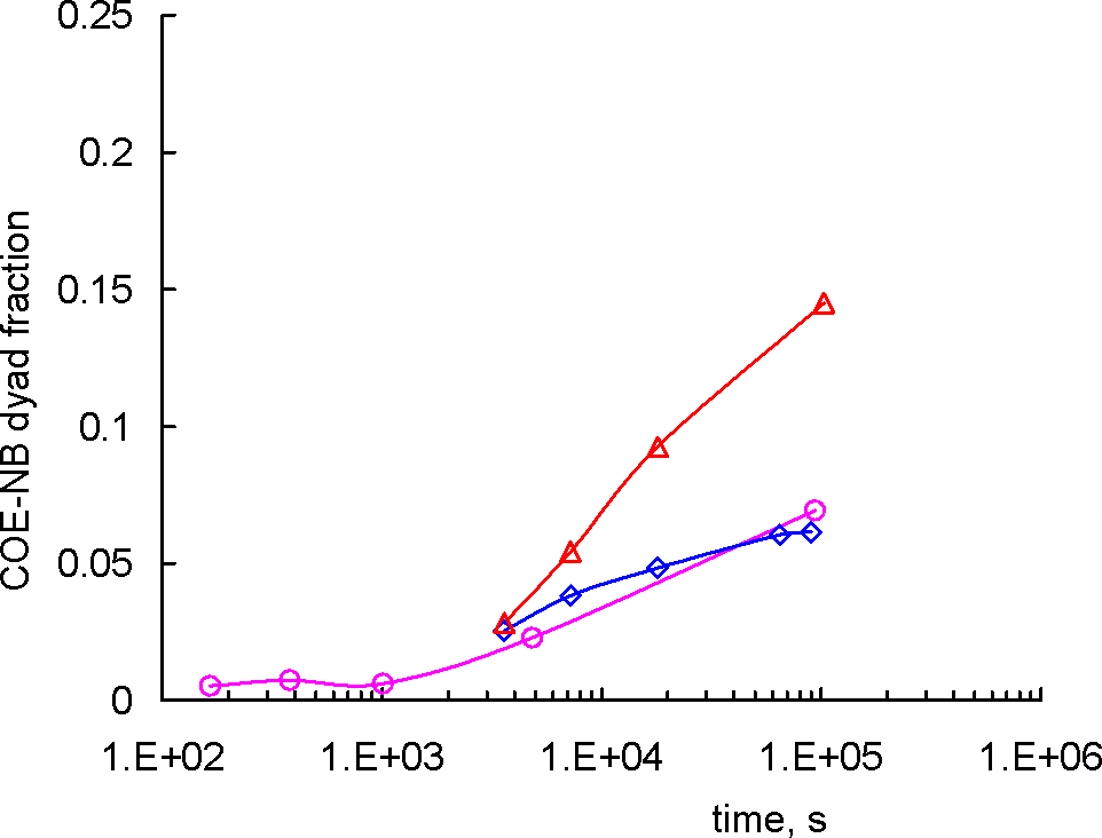
The kinetics of NB-COE dyads formation under mixing conditions for the systems with (red) *c*_in_/*c*_p_ = 1.0 × 10^−2^, [COE]/[NB] = 0.54/0.46; (blue) *c*_in_/*c*_p_ = 3.4 × 10^−3^, [COE]/[NB] = 0.53/0.47; (purple) *c*_in_/*c*_p_ = 1.0 × 10^–2^, [COE]/[NB] = 0.69/0.31. The curves are only for eye guidance.

Before concluding this paper we would like to briefly discuss the role of the polymer/catalyst initial ratio and of the polymer mixture composition. The former parameter determines the final molar mass of the NB-COE copolymer. However, if we consider the dependence of the NB-COE dyad fraction on time (Figure 7), both parameters appear not so important at the early stage. Now it is clear that this stage is associated with the formation of polymer carbenes rather than with the cross-metathesis itself. Later on, the content of alternating dyads grows predictably slower for the system with a lower catalyst loading (cf. the red and blue curves) and for the compositionally asymmetric mixture (cf. the red and purple curves). Note that these experiments were carried out under constant mixing of the reaction media [15], which was impossible for in situ experiments.

## Conclusion

The kinetic data analysis undertaken in the present study makes it possible to outline the cross-metathesis scenario for the mixtures of PCOE and PNB in the presence of the Gr-1 catalyst. Contrary to the situation with a corresponding monomer mixture, where this catalyst first initiates vigorous polymerization of norbornene and only then polymerizes cyclooctene, in the polymer system it first interacts with PCOE and approximately in an hour all Ru-carbenes become bound to PCOE chains. This stage is also characterized by a marked decrease in the average molar mass of the mixture. Then, the cross-metathesis actually starts and it takes about a day to obtain a statistical NB-COE copolymer under chosen conditions, while its molar mass is kept nearly constant. The process is controlled by the slowest elementary reaction, which is the interaction between a [Ru]=PCOE carbene and a double C=C bond in a PNB chain. We suppose that this reaction can be sterically hindered by the bulky structure of a norbornene monomer unit. During the cross-metathesis, [Ru]=PNB carbenes exist at a low concentration but their presence is crucial for the course of the whole process. For developing the cross-metathesis as a new method of obtaining unsaturated statistical copolymers, especially promising for the comonomers with considerably different polymerization rates, it would be interesting to also try a one-pot process, in which case the reaction starts with a monomeric mixture of COE and NB. This could eliminate tedious procedures of homopolymer isolation and purification and allow increasing the concentration of the reacting solution.

## Experimental

### Chemicals

All manipulations involving air- and moisture-sensitive compounds were carried out in oven-dried glassware using dry solvents and standard Schlenk and vacuum-line techniques under argon atmosphere. Monomers, norbornene (Acros Organics) and *cis*-cyclooctene (Aldrich), were dried over sodium, distilled, and stored under argon. The 1^st^ generation Grubbs’ catalyst Cl_2_(PCy_3_)_2_Ru=CHPh (Aldrich) was used without further purification as 0.007–0.077 M solutions in toluene or CHCl_3_. All other reagents and solvents were purchased from Aldrich and used as received or purified according to standard procedures.

### Instrumentation

Nuclear magnetic resonance measurements were carried out at room temperature using a Bruker Avance™ 600 NMR spectrometer operating at 600.22 MHz (^1^H NMR) and 150.93 MHz (^13^C NMR); CDCl_3_ (Aldrich) was used as solvent. Chemical shifts δ were reported in parts per million relative to the residual CHCl_3_ signal as an internal reference standard. Differential scanning calorimetry (DSC) thermograms were recorded on a Mettler TA 4000 system at a rate of 10 °C/min under argon flow of 70 mL/min in the range from −100 °C to 100 °C. The molar mass of the polymers was determined by GPC on a Waters high pressure chromatograph equipped with a refractometric detector and Microgel mix 1–5 μm 300 × 7.8 mm Waters Styragel HR 5E column, with toluene for PNB and NB-COE copolymers and tetrahydrofurane for PCOE as a solvent, the flow rate of 1 mL/min, sample volume of 100 μL, and sample concentration of 1 mg/mL. The molar mass and its dispersity (*Ð*) were calculated by a standard procedure relative to polystyrene standards. Light scattering was studied on a Photocor Complex goniometer equipped with a HeNe laser (a wavelength of λ = 633 nm, an intensity of 25 mW) as a light source. The scattering angle θ was varied in the range 30–150°. In static experiments, the total scattering intensity was measured. In dynamic experiments, the time cross-correlation function *g*_2_ of the scattered-light intensity fluctuations was determined with a 288-channel Photocor-FC correlator board and treated with the Alango DynaLS software through the inverse Laplace transform method to yield the hydrodynamic radius distributions. Prior to measurements, the solutions in CHCl_3_ were filtered through a polytetrafluoroethylene membrane with the pore diameter of 0.22 μm.

### Polymer synthesis (typical)

**Polyoctenamer (PCOE):** C*is*-cyclooctene (3.58 g, 32.6 mmol) was added to the 1^st^ generation Grubbs’ catalyst (38.3 mg, 0.0465 mmol) solution in CH_2_Cl_2_ (12.2 mL) prepared in a round-bottom glass flask (50 mL) equipped with a magnetic stirrer under inert atmosphere at 20 °C. The polymerization was stopped by the addition of 0.3 mL of ethyl vinyl ether after 2 h. The polymers were precipitated in a 0.1% acetone solution of an antioxidant 2,2’-methylenebis(6-*tert*-butyl-4-methylphenol) (**1**), decanted, washed with several portions of the same solution, and dried under reduced pressure at room temperature until constant mass. The yield was 2.72 g (76%). Polymer (1 g) was dissolved in 0.4% THF solution of HCl (30 mL), stirred for 4 h and precipitated in a 0.1% ethanol solution of an antioxidant **1**, decanted, washed with several portions of the same solution, and dried under reduced pressure at room temperature until constant mass. Immediately before the cross-metathesis, 0.9 M polymer solution in CHCl_3_ was passed through a column with SiO_2_ (SiO_2_/PCOE 8:1, w/w) and precipitated in ethanol, decanted, washed with several portions of ethanol, and dried under reduced pressure at room temperature until constant mass. *M*_n_ = 120000 g/mol, *Ð* = 1.8, *T*_g_ = −79 °C, *T*_m_ = 44 °C, *trans*-68%.

**Polynorbornene (PNB):** 4.0 mL of a 3.2 M solution of norbornene (1.21 g, 13 mmol) in toluene was added to the 1^st^ generation Grubbs' catalyst (43 mg, 0.052 mmol) solution in toluene (12.3 mL) prepared as described above at 20 °C. The polymerization was stopped by the addition of 0.4 mL of ethyl vinyl ether after 1 h. The polymers were precipitated in a 0.1% ethanol solution of antioxidant **1**, decanted, washed with several portions of the same solution, and dried under reduced pressure. The polymer was twice reprecipitated in ethanol from toluene solution and dried under reduced pressure at room temperature until constant mass. The yield was 1.20 g (99%). PNB was purified with HCl solution in THF and column chromatography (SiO_2_) as described above. *M*_n_ = 60000 g/mol, *Ð* = 2.6, *T*_g_ = 39 °C, *trans*-88%.

Other thermal characteristics as well as the NMR spectra details of the synthesized PCOE and PNB and NB-COE copolymers are given in our previous paper [15].

### Monitoring Gr-1 – polymer interaction

**Figure 8:**
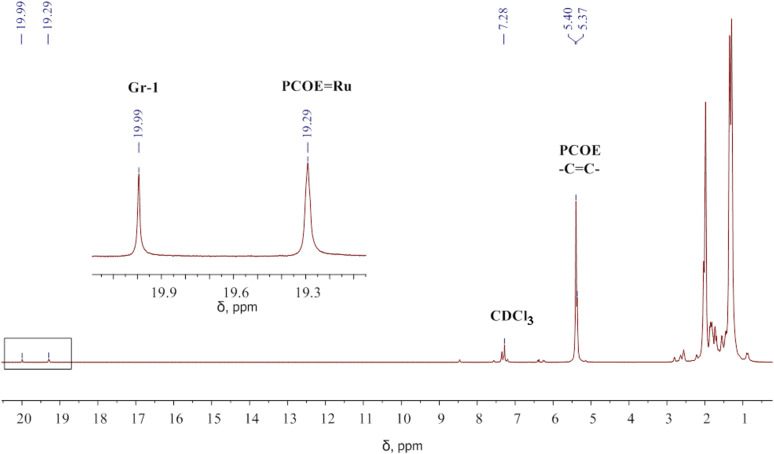
The ^1^H NMR spectrum recorded after 10 min of the reaction between PCOE and Gr-1 at the initial concentration ratio of 20:1 mol/mol in CDCl_3_. Carbene signals (19.99 ppm for [Ru]=CHPh and 19.29 ppm for [Ru]=PCOE) are enlarged in the inset.

PCOE (44.4 mg, 0.35 mmol) and CDCl_3_ (0.46 mL) were placed into a Young’s NMR tube under Ar atmosphere for 24 h with periodic mixing until a homogenous polymer solution was obtained. The mixture was degassed three times by using the freeze-pump-thaw technique before the 0.08 M separately prepared solution of Gr-1 in CDCl_3_ (0.25 mL, 16.4 mg, 0.0199 mmol) was added to the frozen polymer solution. The mixture was melted, mixed, and immediately put into the NMR spectrometer at 20 °C. A typical ^1^H NMR spectrum is shown in Figure 8.

**Figure 9:**
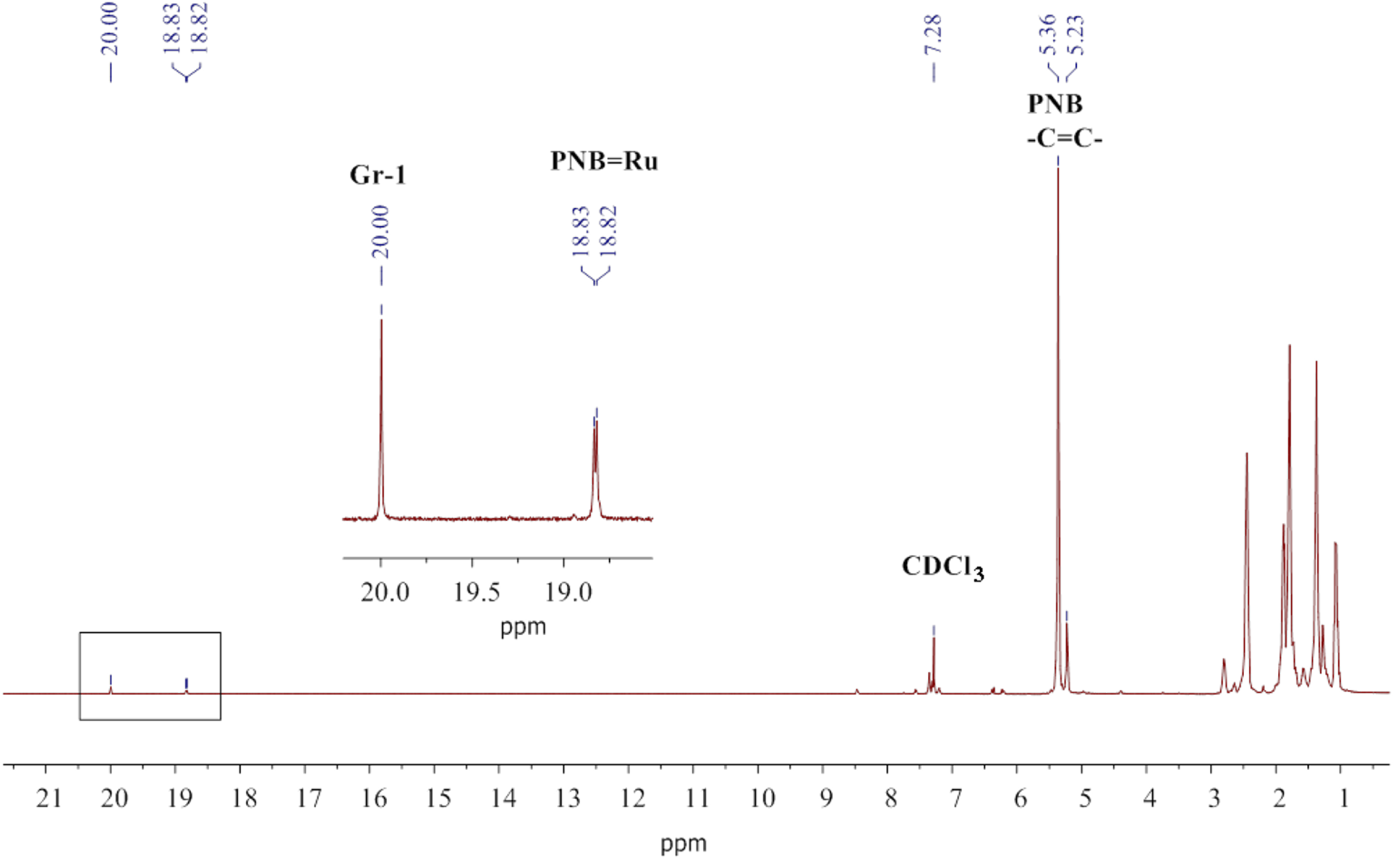
The ^1^H NMR spectrum recorded after 653 min of the reaction between PNB and Gr-1 at the initial concentration ratio of 20:1 mol/mol in CDCl_3_. The carbene signals (20.00 ppm for [Ru]=CHPh and 18.82, 18,83, 18.94 ppm for [Ru]=PNB) are enlarged in the inset.

PNB (33 mg, 0.35 mmol) and CDCl_3_ (0.35 mL) were placed into a Young’s NMR tube under Ar atmosphere for 24 h with periodic mixing until homogenous polymer solution was obtained. The mixture was degassed three times by using the freeze–pump–thaw technique before the 0.08 M separately prepared solution of Gr-1 in CDCl_3_ (0.22 mL, 14.4 mg, 0.0176 mmol) was added to the frozen polymer solution. The mixture was melted, mixed, and immediately put into the NMR spectrometer at 20 °C. A typical 1H NMR spectrum is shown in Figure 9. After a reaction time of 24 h, the molar mass of PNB dropped to *M*_n_ = 11200 g/mol, *Ð* = 1.8.

### Monitoring the cross-metathesis

**Figure 10:**
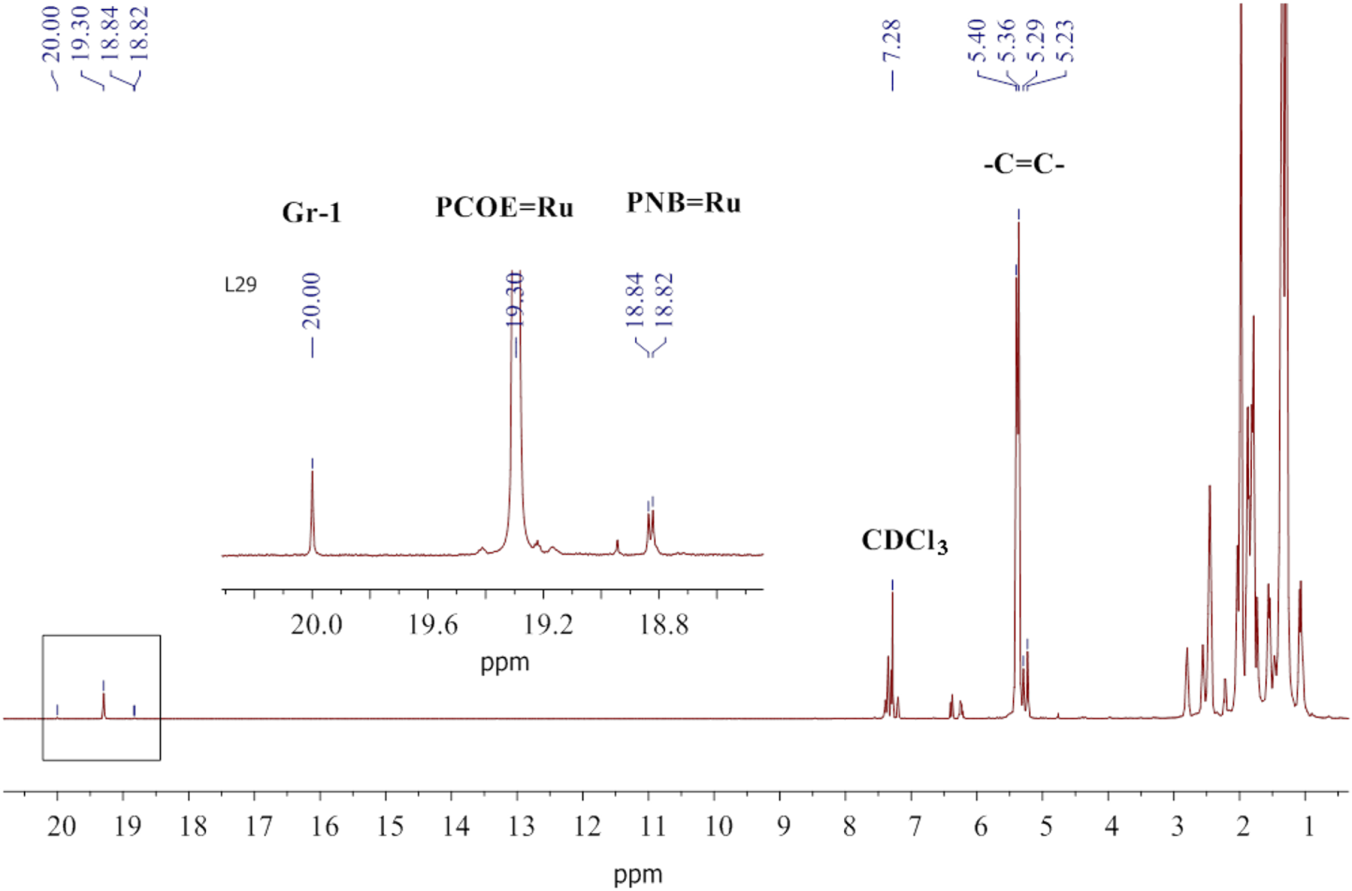
The ^1^H NMR spectrum recorded after 24 h of the reaction between PCOE, PNB, and Gr-1 at the initial concentration ratio of 10:10:1 mol/mol in CDCl_3_. The carbene signals (20.00 ppm for [Ru]=CHPh, 19.30 for [Ru]=PCOE, and 18.82, 18.83, 18.94 ppm for [Ru]=PNB) are enlarged in the inset.

**In situ ^1^H NMR:** PNB (26 mg, 0.25 mmol), PCOE (22 mg, 0.25 mmol), and CDCl_3_ (0.38 mL) were placed into a Young’s NMR tube in Ar atmosphere for 24 h with periodic mixing until homogenous polymer solution was obtained. The mixture was degassed three times using the freeze–pump–thaw technique before the 0.063 M separately prepared solution of Gr-1 in CDCl_3_ (0.37 mL, 20 mg, 0.023 mmol) was added to the frozen polymer solution. The mixture was melted, mixed, and immediately put into the NMR spectrometer at 20 °C. A typical ^1^H NMR spectrum is shown in Figure 10. After 24 h of the reaction, an amorphous NB-COE copolymer of *M*_n_ = 7000 g/mol, *Ð*=1.6, *T*_g_ = −53 °С was formed.

**Figure 11:**
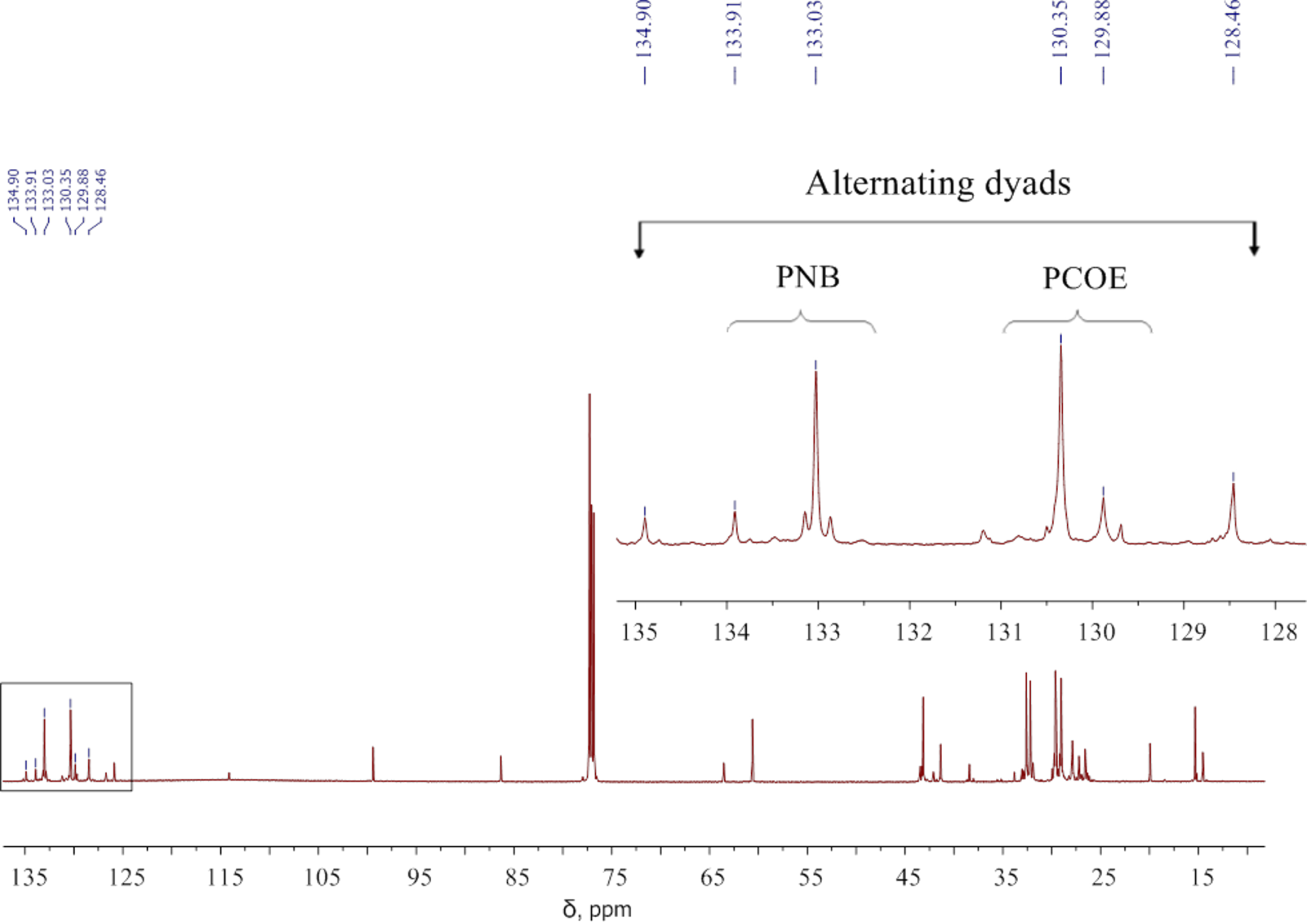
The ^13^C NMR spectrum recorded after 8 h of the reaction between PCOE, PNB, and Gr-1 at the initial concentration ratio of 10:10:1 mol/mol in CDCl_3_. The region of C=C signals including those from alternating NB-COE dyads (128.5, 134.90 m ppm) is enlarged in the inset.

**Ex situ ^13^C NMR:** PNB (156 mg, 1.68 mmol) and PCOE (182 mg, 1.68 mmol) were dissolved in CHCl_3_ (3 mL) in a round-bottom glass flask (25 mL) under inert atmosphere at 20 °C. Then a 0.031 M solution of Gr-1 (2.16 mL, 142.3 mg, 0.173 mmol) in CHCl_3_ was added. Samples for NMR analyses were obtained by adding an aliquot (0.9 mL) of the reaction mixture to 0.2 mL of ethyl vinyl ether, stirred for 30–40 min at ambient temperature, and concentrated in vacuum, after that CDCl_3_ was added. For DSC and GPC measurements, the copolymers were precipitated in ethanol and dried as described above. A typical ^13^C NMR spectrum is shown in Figure 11.
